# Role of antigen specific T and B cells in systemic and mucosal immune responses in ETEC and *Shigella* infections, and their potential to serve as correlates of protection in vaccine development

**DOI:** 10.1016/j.vaccine.2019.03.040

**Published:** 2019-06-20

**Authors:** Sachin Mani, Franklin R. Toapanta, Monica A McArthur, Firdausi Qadri, Ann-Mari Svennerholm, Bert Devriendt, Armelle Phalipon, Daniel Cohen, Marcelo B Sztein

**Affiliations:** a PATH, Washington, DC, United States; b Center for Vaccine Development and Global Health, University of Maryland School of Medicine, Baltimore, MD, United States; c Infectious Diseases Division, International Center for Diarrheal Diseases Research, Dhaka, Bangladesh; d Department of Microbiology and Immunology, Institute of Biomedicine, University of Gothenburg, Sweden; e Faculty of Veterinary Medicine, Department of Virology, Parasitology, and Immunology, Ghent University, Belgium; f Molecular Microbial Pathogenesis, INSERM U1202, Institut Pasteur, Paris, France; g School of Public Health, Sackler Faculty of Medicine, Tel Aviv University, Israel

**Keywords:** Immunocorrelate, Enterotoxigenic Escherichia coli (ETEC), *Shigella*, T cells, B cells, Mucosal antibodies, IgA, IgG, Correlates of protection (CoP)

## Abstract

The generation of robust systemic and mucosal antibody and cell-mediated immune (CMI) responses that are protective, long-lasting, and can quickly be recalled upon subsequent re-exposure to the cognate antigen is the key to the development of effective vaccine candidates. These responses, whether they represent mechanistic or non-mechanistic immunological correlates ofprotection, usually entail the activation ofT cell memory and effector subsets (T-CMI) and induction of long-lasting memory B cells. However, for ETEC and *Shigella*, the precise role of these key immune cells in primary and secondary (anamnestic) immune responses remains ill-defined. A workshop to address immune correlates for ETEC and *Shigella*, in general, and to elucidate the mechanistic role of T-cell subsets and B-cells, both systemically and in the mucosal microenvironment, in the development of durable protective immunity against ETEC and *Shigella* was held at the recent 2nd Vaccines against *Shigella* and ETEC (VASE) conference in June 2018. This report is a summary of the presentations and the discussion that ensued at the workshop.

## 1 Introduction

Enterotoxigenic *Escherichia coli* (ETEC) and *Shigella* are two-leading attributable bacterial causes of diarrhea in young children and in travelers to endemic regions While improved access to nutrition, general infrastructure improvements and prevention strategies such as water, sanitation and hygiene (WASH) approaches have led to decreases in global diarrheal disease mortality due to ETEC and *Shigella*, disease burden and diarrheal morbidity remain high in resource poor settings in Africa and Asia [1,2]. Prevention of ETEC and *Shigella* infections can be much improved through vaccination, but there are no licensed vaccines for either pathogen [3,4]. Progress has been hampered by the limited knowledge of immunological mechanisms that protect the host against these pathogens and relevant immunological parameters that can predict disease outcome and/or vaccine efficacy [5].

While, in general, mucosal IgA responses to either colonization factors (CFs) or the heat labile toxin have been observed to be associated with protection against ETEC diarrhea and serum IgG responses to either LPS or conserved Ipa proteins to be associated with protection against shigellosis, distinct immunological correlates of protection (CoP) for ETEC and *Shigella* are yet to be identified [6]. To address this need, a workshop entitled, “Role of antigen specific T and B cells in systemic and mucosal immune responses in ETEC and *Shigella* infections, and their potential to serve as correlates of protection in vaccine development” was held at the recent Vaccines against *Shigella* and ETEC (VASE) meeting in June 2018, featuring a panel of experts provided an opportunity to discuss and share advances in *Shigella* and ETEC immunology pertaining to mechanistic observations from natural studies and vaccine trials, identify gaps and propose strategies to uncover CoP for these two major pathogens. Five presentations (three in ETEC and two in *Shigella*) featured current knowledge on humoral and T-CMI responses to *Shigella* and ETEC during natural infection and vaccination. A discussion followed on strategies toward identification and selection of immunological correlates that would transcend all age groups, immune status and vaccination modalities. Specific recommendations proposed to facilitate “the path forward” included designing appropriate controlled human infection model (CHIM) studies that would mimic various target populations, as well as utilizing tools to better decipher the immune response.

## 2 Correlates and mechanistic observations from natural ETEC infections and ETEC vaccination [Drs. F. Qadri, icddr,b and AM. Svennerholm, University of Gothenburg]

Protection against ETEC is most likely provided locally at the site of ETEC colonization in the small intestine by secretory IgA antibodies against ETEC colonization factors (CFs), the heat labile toxin (LT) and potentially other protective ETEC surface antigens. Observations from natural infection studies have demonstrated relationships between pre-immune titers (antibody levels in serum and/or mucosal specimens) against ETEC antigens and incidence of diarrheal disease caused by ETEC expressing the corresponding antigens.

Data from birth cohort studies have shown that a repeat episode of diarrhea or infection by the homologous CF type was uncommon in children with symptomatic or asymptomatic infections by CFA/I, CS1 plus CS3, CS2 plus CS3, or CS5 plus CS6 strains [7]. In ETEC challenge studies, increased levels of plasma IgA and IgG antibodies to LTB, CFA/I and CS6 were observed at day 7 after ETEC infection with concomitant increases in circulating antibody secreting cells (ASCs) of IgA and IgG isotypes for these three antigens [8,9] However, while natural infection with ETEC leads to increases in LTB, CFA/I and CS6 specific antibodies and antigen specific ASC at early convalescence, only increases in LTB and CFA/I specific responses were observed at late convalescence LTB and CFA/I specific memory B cell responses were also elevated in patients with ETEC diarrhea. When antibody avidity index (AI) was correlated with memory B cell (B_M_) responses in patients infected with ETEC, IgA specific AI strongly correlated with IgA-MBC for both CFA/I and CS6 antigens [8]. Good correlations were observed between gut homing ASCs and specific antibody isotypes [9].

An orally administered inactivated ETEC vaccine candidate consisting of a combination of recombinantly produced CTB (rCTB) and formalin-inactivated ETEC bacterial strains expressing the CFs CFA/I and CS1-CS5, as well as some of the most prevalent ETEC O-antigens, the rCTB-CF ETEC vaccine, was developed [10] and successfully evaluated in Phase I and II trials in adult volunteers in Sweden, Bangladesh and Egypt, where the vaccine was well tolerated and elicited mucosal immune responses against the different vaccine CFs in >70% of the vaccinees [11–13]. The protective efficacy of the vaccine was evaluated in two large placebo-controlled Phase III trials in American travelers going to Mexico and Guatemala. In these studies, the vaccine was shown to provide protection against moderate to severe diarrhea (MSD) caused by ETEC strains expressing vaccine preventable outcomes (VPOs), i.e., vaccine related antigens [14]. When anti-CTB serum IgA titers induced by the vaccine at arrival in Guatemala was evaluated as a marker for reduced risk for developing traveler’s MSD, an anti-CTB titer greater than 360 was observed to reduce the risk of developing MSD. In a similar study in Egypt, higher anti-CFA/I IgG titers in serum of rCTB ETEC vaccinated Egyptian children less than 18 months correlated inversely with the development of ETEC CFA/I diarrhea [15]. In a subsequent Phase I study in adult Swedish volunteers, the improved oral inactivated ETEC vaccine, ETVAX consisting of recombinant ETEC strains overexpressing ETEC CFs and an LT toxoid, in the presence of the double mutant (dm)LT adjuvant was evaluated [16]. In this study, as many as 83% of the vaccinees given two oral doses of ETVAX+ 10 mg dmLT responded robustly to all five primary vaccine antigens (CFA/I, CS3, CS5, CS6 and LTB) [16]. When LTB responses were further dissected, 97% of the responders generated an over 15-fold rise in mucosal IgA responses and over 80% had neutralizing Ab to the LT toxin. When previously vaccinated volunteers were recalled and given an oral vaccine boost one to two years later, responses against CFs expressed by the vaccine were much higher than in naive individuals given a single dose of the vaccine [17]. The ETVAX+ 10 mg dmLT also induced strong IFN-γ and IL-17AT cell responses to all vaccine CFs and LTB; depletion experiments verified that these responses were primarily produced by vaccine + dmLT induced CD4 + T cells [Lundgren et al., unpublished]. Observations from a descending age study in Bangladesh demonstrated that 100% of adults responded with mucosal immune responses to the five primary vaccine antigens [18] and that children down to 6 months of age responded with significant mucosal fecal and or ASC/ALS responses to all those antigens [Svennerholm et al., unpublished data]. In summary, birth cohorts, CHIM and vaccine studies all point to an important role of anti-CF and anti-LT mucosal immune responses in protection against ETEC associated diarrhea.

## 3 ETEC infection and vaccination: insights from a pig model [Dr. Bert Devriendt, Ghent University]

Natural ETEC infections in livestock such as pigs cause neonatal and post-weaning diarrhea, resulting in severe economic losses to the pig industry due to mortality, reduced growth rates and increased medication [19]. In addition to the physiological and immunological similarities between humans and pigs, ETEC pathogenesis in swine is identical to that in humans. This attribute makes swine an ideal large animal model to study host-pathogen interactions in the gut as well as evaluate vaccination strategies to prevent pediatric ETEC infections [20]. Like humans, involvement of fimbriae in adhesion to the small intestinal epithelium, make them ideal targets for vaccine strategies against porcine ETEC strains. The most prevalent porcine ETEC strains are those that possess F4 and F18 fimbriae, whose receptors are aminopeptidase N (APN) and blood group ABH type 1 carbohydrates [21,22]. These receptors govern susceptibility to ETEC infection and are also required to mount robust intestinal immunity upon oral vaccination with fimbriae-based vaccines.

Clearance of ETEC infections in pigs is associated with the induction of fimbriae-specific secretory IgA (SIgA) in intestinal tissues, such the small intestinal *lamina propria* and mesenteric lymph nodes [23]. Induction of these SIgA probably involves fimbriae-specific intestinal Th17 cells [24]. This mechanism was evaluated in a few oral immunization studies with purified fimbriae that in susceptible piglets triggered local fimbriae-specific SIgA, which provide protection against challenge infection with the homologous ETEC strain [25,26]. This was associated with the induction of fimbriae-specific IL17-producing lymphocytes in blood and intestinal tissues [24].

In piglets, passive protection afforded by either maternal antibodies or SIgA antibodies in feed have helped to overcome some of the constraints posed by oral vaccination strategies on an immature immune system. In some instances, preexisting maternal antibodies at the time of oral vaccination, help to boost vaccine efficacy. Upon withdrawal of milk from piglets, fimbriae-specific maternal antibodies in serum might enhance the efficacy of oral vaccination with purified fimbriae in these piglets [27]. In other instances, artificial strategies to prolong passive immunity such as delivery of fimbriae-specific SIgA molecules produced in plant seeds and given to the piglets in their feed provided protection to the piglets against challenge infection with ETEC – helping them tide over the time needed by their immune systems to mount an active response [28]. Use of these plantibodies have helped to bridge the gap between passive and active immunity.

In summary, fimbriae-specific receptors are necessary for susceptibility to ETEC infection and to trigger robust local SIgA responses upon oral vaccination with fimbriae-based vaccines, which provide protection to piglets against ETEC-induced diarrhea.

## 4 Review of mechanistic T follicular/helper (T_FH_) responses across two studies – ETEC challenge and ETVAX vaccination [Drs. Monica M. McArthur and Marcelo B. Sztein, CVD, University of Maryland School of Medicine]

The third presentation of the workshop focused on T-CMI responses in volunteers challenged with wild-type ETEC strain H10407. Since ETEC is a non-invasive luminal pathogen, mucosal humoral responses are believed to be a major contributor to protection. Given the key role that CD4+ T cells play in the development, enhancement and maintenance of antibody responses, it is critical to also understand the role of this critical effector arm of the immune response including the production of key Th1 cytokines such as IL-2, IFN-γ, IL-17A, in response to antigenic stimulation [29]. Circulating T follicular/helper (cT_FH_) cells are a key CD4+ subset characterized by the expression of CXCR5 that plays a critical role in promoting humoral responses [30]. In general, development of systemic and mucosal antibody responses to ETEC are driven by effector immunity components such as CD4+ and cT_FH_ activation, cytokine production and expression of homing molecules. Peripheral blood mononuclear cells (PBMC) collected both pre- and post-challenge during a recent ETEC CHIM study were evaluated for CD4+, and, cT_FH_ responses following challenge with wild-type (wt) ETEC. CD4+ T cell responses to *in vitro* stimulation with ETEC antigens pre- and post-challenge and their association with longterm B memory (B_M_) responses were also evaluated. Increased production of CFA/I-specific TNF-α and IL-2 by multifunctional CD4+ T cells 3 days after challenge were observed in volunteers who did not develop disease (Resistant) following challenge [31]. Significantly higher levels of integrin α4p7 expression, a molecule associated with homing to the gut, the site of initial encounter with ETEC [32], were also observed in CD4+ T cells from volunteers who were Resistant, suggesting that these CD4+ cells possess the ability to home to the site of pathogen encounter and may play a role in protection. The higher expression of integrin α4p7 in cT_FH_ of Resistant volunteers, at day 3 post challenge, correlated with lower cumulative stool volumes and with ETEC-specific IgA B_M_ responses [31]. These results support the notion that interaction between antigen-specific cT_FH_ and their B cell counterparts in the gut-associated lymphoid tissues such as Peyer’s patches and/or mesenteric lymph nodes, results in antigen-specific B_M_ responses which may confer long-term protection in humans.

Very recently, the observations that exposure to ETEC results in the induction of cT_FH_ were confirmed in a study involving Swedish adults who received 2 doses of the oral inactivated ETVAX ETEC vaccine [33]. These studies showed enhanced expression of ICOS, IL-21, Th17 markers and integrin p7 by activated cT_FH_ cells in antibody secreting cell (ASC) “responders” within 1 week after primary vaccination. Moreover, co-culture experiments showed that cTFH cells promoted post-vaccination memory B cells to differentiate and secrete higher levels of LTB-specific IgA antibodies. Taken together, these two studies suggest that activated cT_FH_ cells are mobilized into blood after oral vaccination or wild-type ETEC challenge and may be used as biomarkers of ETEC vaccine specific mucosal memory in humans. Thus, further exploration of T cell responses, particularly cT_FH_, and their role in protection against ETEC are warranted.

## 5 Serum IgG antibodies to *Shigella* LPS – a correlate of protection against shigellosis (Drs. Daniel Cohen, Tel Aviv University and Armelle Phalipon, Institut Pasteur)

Observations from natural and experimental infections with *Shigella* have shown that these exposures confer serotype specific immunity to subsequent *Shigella* exposure of the same serotype in around 70% of the individuals [34–38]. This protection lasts for around two years and suggests that the O-specific polysaccharide of *Shigella* LPS is the protective antigen against shigellosis [36–38]. In observational sero-epidemiological studies, pre-existing anti-LPS IgG antibodies to *S. sonnei* or S. *flexneri* 2a decreased the likelihood of subsequent shigellosis with the homologous, but not the heterologous *Shigella* species, supporting previous data on occurrence of serogroup-specific immunity after shigellosis [39,40]. These findings are supported by data in which the presence of serum IgA and IgG antibodies prior to experimental challenge with virulent *S. sonnei* was correlated with protection against illness [41].

In view of the data showing that serum IgG to *Shigella* LPS were associated with resistance to shigellosis caused by the homologous serotype, experimental *Shigella* conjugate vaccines were generated at the National Institutes of Health (NIH) by John Robbins and Rachel Schneerson using detoxified O-specific polysaccharide from *S. sonnei* or from *S. flexneri 2a* covalently bound to the recombinant *Pseudomonas* exoprotein A (rEPA) (*S. sonnei*-rEPA, *S. flexneri* 2a - rEPA) [42,43]. The *S. sonnei-rEPA* conjugate vaccine was found to elicit stronger increases in the titers of serum IgG antibodies against homologous LPS, when compared to natural infection [44,45]. Data from two efficacy trials of a *S. sonnei* conjugate vaccine and from *S. sonnei* human challenge studies also confirm a strong association between the level of serum IgG antibodies to *Shigella* LPS and reduced risk of homologous naturally occurring or induced shigellosis. In the 1st field efficacy trial, 74% protection was observed in young adults with a strong increase in serum IgG titers following the single injected dose of the vaccine [44]. In a subsequent study in children aged one to four years, protection from *S. sonnei* shigellosis was age dependent after two doses of the S. *sonnei* conjugate vaccine and correlated with the levels of anti-*S. sonnei* LPS IgG. In children aged three to four years, protection was as high as 71% that dropped to less than 5% in children less than two years of age [46]. When functionality of the serum antibodies was evaluated in adults who received the *S. sonnei*- rEPA vaccine, 78% of the responders had specific *S. sonnei* IgG exhibiting strong serum bactericidal activity. A synthetic carbohydrate-based conjugate vaccine, SF2a-TT15 against S. *flexneri* 2a was designed and constructed at Institut Pasteur [47,48] aiming to obtain a more immunogenic vaccine conferring protection against shigellosis in children younger than 3 years of age. In a phase I study recently completed in Israel, the SF2a-TT15 conjugate vaccine induced a stronger serum IgG response as compared with the 1st generation of detoxified O-specific polysaccharide-based S. *flexneri* 2a- rEPA conjugate [49, Cohen D et al., unpublished data]. These data in humans with the SF2a-TT15 conjugate corroborate previous findings in mice showing that *Shigella* conjugates with shorter synthetic saccharides can be stronger inducers of anti-LPS IgG than conjugates incorporating the whole O-SP of the same strain [49]. The protective efficacy of the SF2a-TT15 conjugate and the role of serum IgG raised against S. *flexneri* 2a LPS induced by the vaccine as correlates of protection will be further evaluated in a controlled human infection model.

## 6 Review of mechanistic observations from *Shigella* vaccine and challenge studies [Drs. Franklin Toapanta and Marcelo B. Sztein, CVD, University of Maryland School of Medicine]

The final presentation focused on T-CMI responses in volunteers challenged with *Shigella* as well as those that were vaccinated with live-attenuated *Shigetta* vaccine candidates that mimic various aspects of the initial steps of infection in humans. *Shigella*, being an intracellular pathogen not only infects gut epithelial cells, but also gut immune cells such as macrophages, T cells and B cells [50–55]. Macrophages and B cells are antigen-presenting cells, which can efficiently stimulate and activate T cells. Therefore, *Shigella* infections are expected to induce T-CMI; however, very little data regarding these immune responses are available. Previous studies had provided indirect evidence of these responses. For example, rectal biopsies of acutely infected or convalescent human volunteers showed activation of intraepithelial T cells and macrophages. Moreover, IL-1α, IL-1β, IFN-γ, TNF-α, IL-1Rα, IL-6, IL-8, IL-4, IL-10 and TNF-β were detected in rectal tissues [56]. In patients with *S. dysenteriae* 1 infections, IFN-γ was identified in rectal tissues and the levels of this cytokine were higher in convalescent volunteers than in those with acute disease. Moreover, immunity to shigellosis correlated with up regulation of IFN-γ and IFN-γR [57]. Given the limited availability of specimens from *Shigella* CHIM studies, live-attenuated *Shigella* vaccine candidates, which mimic various aspects of the initial steps of infection in humans, have provided opportunities to explore induction of T-CMI in peripheral blood (PBMC and sera). Vaccines such as SFL1070 (*S. flexneri* 2a; *ΔaroD)* induced cells that produced IFN-y upon stimulation with *S. flexneri* 2a polysaccharide antigen or Ipa [58]. Studies using other vaccine candidates, such as CVD 1203 (*Δaro*A, *Δvir*G), SC595 (*Δstx*A), WRSS1 (*Δvir*G), and CVD 1208S (*Δgua*BA, *Δsen, Δset*) have also shown the induction of cytokines either in sera or by PBMC upon *ex-vivo* stimulation with *Shigella* antigens [59–62].

Despite all these advances, the identity of the T cell subsets producing the cytokines at the local (intestine) and systemic levels remains rather limited. A recent report using CVD 1208S, showed that upon 3 oral vaccine doses, ~70–90% of vaccinees exhibited T-CMI responses [63]. T-CMI was detected in both CD8+ and CD4 + T cells. T effector memory (T_EM_) and central memory (T_CM_) subsets were the main cytokine producers and CD107a-expressors. T cells capable of simultaneously expressing multiple functions (e.g., producing more than one cytokine and/or expressing defined activation markers such as CD107a), are dubbed multifunctional (MF). The presence of these cells has been associated with optimized effector functions, and protection from disease in multiple systems [64,65]. Multifunctional (MF) cells were also detected in CD8 T_EM_ and cells with 2-3 functions were the most abundant. Moreover, the cells that produced higher levels of cytokines, also expressed higher levels of integrin α4β7. These data expanded on the previous knowledge that *Shigella* induces T-CMI and demonstrated that various CD8+ and CD4 T cells subsets were responsible for producing cytokines and upregulating CD107a. Despite these observations, the lack of CHIM *Shigella* studies were T cell subsets were evaluated that makes it impossible, at present, to correlate these markers with protection from disease. Thus, vaccination followed by challenge studies are urgently needed to address these critical questions. Of note, the assay to determine T-CMI initially developed by conventional flow cytometry has been modernized to take advantage of mass cytometry, a platform that allows evaluation of a wider variety of cell subsets. The current mass cytometry panel for T-CMI is composed of 38 unique makers to study not only CD4 and CD8 T effector memory subsets, but also cT_FH_ cells and other subsets. Of note, expanding T-CMI studies to include cT_FH_ cells is crucial since cT_FH_ drive the development of B_m_ cells, and consequently effective humoral vaccine responses.

Immunization with live attenuated *Shigella* vaccine candidates elicited strong IgG and IgA responses to *Shigella* LPS and IpaB as well as IpaB-specific B memory cells [66–68]. However, the methodologies available at the time that these trials were performed did not allow for comprehensive characterization of these responses. As mentioned above, novel methodologies such as mass cytometry are currently being used to identify antigen-specific B cells that bind *Shigella* and other enteric bacterial pathogens with high avidity. In a proof of principle experiment, when PBMC from a wild-type *Salmonella* Typhi challenge study (S. Typhi CHIM) were studied using multiparametric analysis, the percentage of *S.* Typhi-specific B cells in volunteers who developed typhoid disease upon challenge showed distinct differences in phenotypic markers, compared to pre-challenge levels [Toapanta F, Sztein M et al., unpublished data]. Dimensionality reduction algorithms, such as t-Distributed Stochastic Neighbor Embedding (t-SNE), allowed for the identification of clusters of cells that were part of the B cell naive subset and that had diverse expression of multiple markers (e.g., CD40, CD38, CD95, CD69, CXCR5) depending on disease development. Mass cytometry can be similarly employed in *Shigella* CHIM studies, to identify antigen-specific B cell subsets that might be associated either with disease protection or disease susceptibility.

## 7 Discussion

Following the presentations, discussions revolved around the difficulties of defining immune correlates of protection and outlining what are the best CoP currently available. This was followed by an overview of the attributes that an immune correlate must possess as well as the endpoint that is being targeted. The recommendations from participants in the correlates of enteric vaccine-induced protection conference helped to guide the panel discussion [69–71,6].

In reviewing key points from the five talks, it was observed that there was a general agreement that in ETEC, protection is most likely provided by SIgA antibodies against CFs, LT (and potentially other protective antigens) produced locally in the small intestine, whereas in the case of *Shigella*, serum IgG responses to *Shigella* LPS appear to correlate with protection. However, these responses varied widely among bacterial strains and serotypes, vaccine type, adjuvants used, route of immunization, age, nutritional and immune status. What constitutes protection has also been difficult to be accurately defined in diarrheal diseases – is it protection against infection or disease? Even when considering disease prevention, there is a wide range of definitions, from aiming to curtail severe disease to targeting any/most disease manifestations.

In many instances, serological measures of immunogenicity have not correlated with protection. For instance, no correlations were observed between antibodies to LPS present at the time of challenge and resistance to typhoid fever upon exposure to wt *S.* Typhi [72]. In contrast, T-CMI, especially multifunctional CD8+T_EM_ responses correlated with protection upon challenge with *S.* Typhi, highlighting the need for a better understanding of T-CMI and B memory responses to have a more complete view of the mechanisms that could be involved in protection [73]. However, *S.* Typhi, *Shigella* and ETEC are Gram negative bacteria with very different pathogenic mechanisms; thus, caution should be exercised when attempting to equate CoP between these enteric bacterial infections. While there was consensus that it is important to dissect and understand the role of memory B cells and cT_FH_ cells in driving protective responses, an immune correlate should be easily measurable, and efforts should be directed to identify a threshold of antibody (and isotype) levels that correlate with protection. While this attribute, in general, could be extremely useful in defining a CoP early after vaccination, defining the role of B and T CMI memory in driving serological and mucosal immune responses could be very important in helping to predict the longevity of protective responses induced by vaccination and/or exposure to the pathogens.

Limitations of current assay methodologies in accurately measuring pathogen specific mucosal immune response is a challenge that must be addressed. For instance, in ETEC, anti-CF and anti-enterotoxin IgA responses are localized in the lumen of the small intestine, making it difficult to sample and accurately measure. Whilst Antibodies in Lymphocyte Supernatants (ALS) assays measuring IgA antibodies in supernatants from cultured PBMC are likely to be reflective of local mucosal IgA responses, this procedure is labor intensive and difficult to scale up for field studies. On the other hand, measuring IgA in fecal extracts, while easier to perform, has inherent variability that needs to be extensively standardized. These challenges restrict both assays to very few specialized laboratories [6].

## 8 Recommendations

Following the broad ranging discussion, the panel, as well the audience participants emphasized the need for additional opportunities to interact and exchange data, information and materials with the goal to facilitate identifying CoP that support vaccine development and licensure for ETEC and *Shigella* vaccines. A few specific recommendations were proposed towards achieving this goal:

- Design targeted CHIM studies using a variety of vaccine types, adjuvants and routes of immunization that would afford the evaluation of both humoral and cellular responses and to correlate the observed responses with protection from disease.
- Design and perform sero-epidemiological studies in children and adults in highly endemic regions to examine candidate pre-existing immunological parameters as correlates of a reduced risk to develop homologous disease under natural conditions of exposure.
- Expand the technologies to access mucosal tissue and fluid samples in the appropriate gut segments for the bacterial infection being studied to help define CoP in mucosal tissues.

In conclusion, defining correlates of protection are a key requirement to advance the development of vaccines against ETEC and *Shigella.* Evaluation of these responses with selected vaccine candidates should then be performed at the appropriate time points in vaccine trials in endemic areas.
